# Bridging corpus diagnostics and EAP pedagogy: A corpus-driven study on lexical hedges in Chinese scholars’ spoken academic English

**DOI:** 10.1371/journal.pone.0353106

**Published:** 2026-07-06

**Authors:** Yinchun Wang, Yang Yu, Hanlin Song, Yongkang Wu, Lexuan Lin

**Affiliations:** 1 School of Foreign Languages, Dalian Maritime University, Dalian, Liaoning, China; 2 School of Foreign Languages, Hunan University, Changsha, Hunan, China; UKM: Universiti Kebangsaan Malaysia, MALAYSIA

## Abstract

As the internationalization of higher education intensifies — particularly under China’s “Double First-Class” initiative — Chinese scholars are increasingly expected not only to publish in English but also to perform effectively in high-stakes spoken genres such as conference talks, keynote lectures, and doctoral defences. Yet pragmatic features such as hedging remain a persistent barrier, often leading to pragmatic failure in real-time interactions. This study addresses this challenge through an integrated approach that couples corpus-based diagnostics with pedagogical design. Specifically, it investigates the use of lexical verb hedges in Chinese scholars’ spoken academic English by comparing a self-compiled Chinese Scholars’ Academic Spoken English Corpus (CASEC; 696,009 words; 279 events) with the Michigan Corpus of Academic Spoken English (MICASE; 1,848,364 words; 152 events). Following Granger’s reappraised Contrastive Interlanguage Analysis 2.0, MICASE is treated not as a native-speaker benchmark but as one Anglo-American reference variety against which typical L2 patterns can be described. Quantitative analysis revealed that Chinese scholars significantly underused individualized stance markers (e.g., I think) and significantly overused collective, consensus-building markers (e.g., We see). Qualitative analysis interprets these patterns through the lens of cross-cultural pragmatics and genre constraints, suggesting a rhetorical strategy oriented toward collective harmony and the diffusion of authorial responsibility. Crucially, the study moves beyond diagnosis to propose a “diagnosis-to-treatment” pipeline, culminating in a step-by-step Data-Driven Learning (DDL) module complete with full classroom worksheets. The pedagogical design aims to raise scholars’ pragmatic awareness and enhance their international academic communication competence.

## 1. Introduction

In global higher education, English is the lingua franca of academic exchange. For scholars worldwide, disseminating research in English is a professional necessity. This reality is particularly acute in China, where the “Double First-Class” initiative aims to elevate universities to world-class status. Consequently, Chinese scholars face pressure not only to publish in high-impact international journals but also to present effectively at international conferences, to defend research in online doctoral committees, and to engage in high-stakes academic discourse in real time.

However, existing literature indicates that linguistic competence, particularly in the domain of grammar and vocabulary, does not automatically translate into pragmatic competence. Academic discourse is no longer viewed merely as an objective conduit for propositional content. It is increasingly conceptualized as a site of social interaction, where scholars employ sophisticated linguistic resources to build arguments, position their claims, and negotiate relationships with their audience [1,2]. Recent systematic reviews highlight that hedging strategies remain a central concern in evolving academic discourse conventions [3]. This interactional dimension of academic communication, or metadiscourse, is crucial for achieving persuasion and gaining community acceptance. It represents the author’s “voice” or “persona”, allowing them to step into their text to manage the flow of information and signal their attitude towards both the content and the reader.

Within the array of metadiscoursal devices, hedges play a pivotal role in constructing an academic stance [1,4,5]. Hedges, particularly epistemic lexical verbs (e.g., think, seem, suggest), are not markers of ambiguity or weakness; rather, they are strategic tools that allow authors to modulate the certainty of their assertions. This modulation serves a dual purpose: it demonstrates academic rigor by acknowledging alternative perspectives and simultaneously performs a crucial interpersonal function by mitigating potential Face-Threatening Acts (FTAs), thereby fostering dialogue [6]. By hedging a claim, a scholar signals humility and openness to debate, which are core values of the scientific community. A scholar who fails to hedge appropriately risks appearing dogmatic, arrogant, or naive, regardless of the empirical validity of their data.

For Chinese scholars, who operate in an EFL (English as a Foreign Language) environment, achieving pragmatic competence in spoken academic English is often more challenging than mastering written norms. While written drafts can be polished by professional editors or refined through multiple rounds of peer review, spoken interactions — such as conference Q&A sessions, lectures, or defenses — require real-time pragmatic processing. The cognitive load of processing L2 syntax and vocabulary often leaves few resources for monitoring pragmatic appropriateness. Consequently, the ability to use hedges appropriately — to balance authorial confidence with academic modesty — is crucial for successful international collaborations. A failure to hedge appropriately can lead to “pragmatic failure” [7], where a scholar might be unintentionally perceived as rude or lacking in critical nuance, potentially jeopardizing their academic reputation.

Yet, pedagogical resources tailored to the specific pragmatic needs of Chinese scholars remain scarce. Most EAP (English for Academic Purposes) training in China relies on intuitive materials or general English textbooks rather than empirical data derived from scholars’ actual performance. This disconnect results in a “pedagogical vacuum,” where scholars are aware of their linguistic deficits but lack the specific tools to remedy them. Furthermore, while stance in academic discourse is a well-researched area [8–11], most prior work has focused on written texts, often at the level of broad metadiscoursal category frequencies. Where spoken stance has been examined [12–14], attention has centred on either adverbial stance markers or pronominal choice in L1-dominant monologic lectures. Comparatively little work has examined the specific, high-leverage phraseology of lexical verb hedges in the real-time spoken output of Chinese faculty-level scholars, and even less has linked such findings to concrete, classroom-ready pedagogical interventions.

Building on — and extending — this mature literature, the present study makes four specific contributions. First, it focuses on fine-grained lexical verb hedges (see, think, assume, consider, and related items) rather than broad metadiscoursal categories. Second, it draws on a newly-compiled, 696,009-word spoken corpus of Chinese faculty-level scholars (CASEC), a population absent from existing learner corpora such as LINDSEI. Third, it uncovers a specific and quantitatively striking “I V that” vs. “We V that” pragmatic mismatch. Fourth, and most importantly, it closes the loop between corpus diagnosis and classroom pedagogy by delivering a fully specified Data-Driven Learning (DDL) module, including sample concordance worksheets, guiding questions, and role-play scenarios (S2 File). Together, these four features — we argue — advance the field from further documentation of cross-cultural stance differences to a practical, reproducible template for pragmatic intervention. This study aims to fill this void by providing a corpus-driven diagnosis of the problems and offering a concrete, data-driven pedagogical solution. In doing so, it contributes to the base of academic knowledge in the specific sense required of original research: it reports new primary data drawn from a scholarly population not previously documented in spoken corpora, new empirical findings derived from those data, and a new, directly reusable pedagogical artefact, rather than replicating analyses already available in the literature.

## 2. Literature review

### 2.1 Stance and metadiscourse in academic communication

The study of academic discourse has shifted decisively from a purely textual focus to a social-interactional one. This perspective views texts as the product of social engagement, where writers and speakers are not just reporting objective facts but are actively “doing” things: persuading, negotiating, and positioning themselves within a community [8]. Hyland’s [1] influential model of metadiscourse provides a robust framework for analyzing this interaction, distinguishing between interactive resources (which organize the text) and interactional resources (which engage the reader). This study focuses on interactional resources, which are the primary means by which authors express their stance — their personal attitudes, evaluations, and commitments to propositional content [9].

Stance is not an optional extra; it is the rhetorical expression of the author’s academic identity and the primary mechanism for building a persuasive argument [10]. As noted by Wang and Jiang [11], successful academic writing requires authors to establish a competent authorial identity by judging the certainty of their knowledge claims while persuading readers of their research contributions. Similar trends have been observed in disciplinary variations and cross-linguistic contexts [12–15]. Furthermore, recent studies have extended this line of inquiry to specific rhetorical moves, such as the presentation of limitations in conclusion sections [16] and intrageneric variations in student writing [17]. In spoken discourse, this requirement is even more pressing, as the speaker is physically present and must manage the audience’s reaction in real-time [9,18].

### 2.2 Hedges as pragmatic devices for stance and politeness

Hedges are a core sub-category of interactional metadiscourse, specifically realizing epistemic stance. Lexical verb hedges, such as think, suggest, assume, and seem, are crucial for allowing speakers to precisely calibrate the level of certainty or commitment to a claim [19]. This calibration is fundamental to academic integrity. Presenting a novel finding as an absolute truth is often seen as unscientific, as science is inherently probabilistic [4,20]. By using a hedge, the scholar presents the claim “cautiously, accurately, and modestly” [4], acknowledging that knowledge is provisional and subject to revision [21]. Recent corpus-based insights further confirm that hedging in spoken discourse is distinct from writing, serving more immediate interpersonal functions such as vaguely referencing shared knowledge or managing dysfluency [22].

The function of hedging is intrinsically linked to politeness theory [6]. The core of this theory is the concept of “face,” or the public self-image that all social actors wish to maintain. Many academic speech acts — such as proposing a new claim that contradicts established theory or refuting an existing hypothesis — constitute Face-Threatening Acts (FTAs). A direct assertion threatens the audience’s face by imposing the speaker’s view. Hedges function as a quintessential negative politeness strategy: by softening the illocutionary force of an assertion (e.g., “I feel your theory may not fully account for...”), the speaker respects the audience’s “negative face” (their desire for autonomy and freedom from imposition). Recently, Ädel [18] proposed viewing these features not just as isolated markers, but as dynamic “moves” within spoken presentations, specifically in how speakers manage real-time interpersonal negotiation.

### 2.3 Cross-cultural pragmatics: The ‘Face’ dilemma

The conventions for signaling stance and politeness are not universal; they are deeply embedded in specific cultural and rhetorical traditions [23,24]. Research in cross-cultural pragmatics has highlighted significant differences between Anglo-American and East Asian rhetorical norms. While Anglo-American rhetoric emphasizes “independence” (negative face) and individual agency, traditional Chinese rhetoric, influenced by Confucian values, often prioritizes “involvement” (positive face) and the maintenance of Mianzi [25,26].

In this cultural framework, modesty (qianxu) is often achieved by minimizing the “self” and emphasizing the “collective” [27]. This leads to an “other-oriented” politeness model where asserting individual authority (via I think or In my opinion) might be viewed as disrupting group harmony or appearing boastful. Instead, strategies that appeal to shared knowledge or collective observation are preferred. This divergence can lead to pragmatic transfer in L2 academic discourse [28,29]. Corpus-based L2 research [30] has provided robust evidence of this phenomenon. Analyses of Chinese L2 academic writing have consistently shown differences in the use of stance resources [31–33]. Studies have shown Chinese EFL speakers may underuse first-person pronouns, reflecting a “collective” stance rather than an “individual” one [34,35]. Recent contrastive studies confirm that Chinese scholars’ spoken discourse exhibits distinct hedging patterns compared to English as a Lingua Franca (ELF) speakers [36,37]. However, much of this existing research has relied heavily on monocausal cultural explanations, often overlooking the influence of genre constraints or the specific cognitive metaphors [38] underpinning these choices. Furthermore, few studies have specifically examined how these cultural scripts manifest in high-frequency lexical verb choices in high-stakes spoken genres like conference presentations.

### 2.4 Data-Driven Learning (DDL) for pragmatic competence

To address the challenges identified by corpus-based L2 research, a pedagogical solution is required that moves beyond prescriptive rules. Data-Driven Learning (DDL), pioneered by Cheng, Warren, and Xun-feng [39], is an inductive, corpus-based pedagogical approach. In a DDL model, learners take on the role of “language detectives,” directly engaging with authentic language data to observe patterns and generalize rules [40]. DDL is particularly well-suited for teaching pragmatic competence, which is highly context-sensitive [41]. The psychological mechanism underpinning DDL is “noticing” [42]. For pragmatic competence to develop, learners must first notice the subtle connections between a specific linguistic form (e.g., I think) and its communicative function. By exposing learners to a high volume of authentic input, DDL facilitates this “consciousness-raising” process [43–45]. Moreover, meta-analyses of technology-enhanced learning suggest that such contextualized instruction is significantly moderated by instructional design [46]. This study argues that DDL is the most effective bridge between corpus diagnostics and classroom practice, transforming abstract research findings into concrete learning materials [47].

### 2.5 Positioning the present study

The study of stance in academic discourse is a mature field, and we wish to be explicit about the debt our work owes to this substantial body of research. In particular, three strands of prior work are especially pertinent. Fortanet-Gómez [48] provides a foundational analysis of the reference and functions of we in L1 university lectures, establishing that inclusive we plays a central organizational and interactional role in L1 spoken academic English. Gablasova, Brezina, and McEnery [49] offer an influential methodological framework for interpreting L1/L2 corpus-frequency contrasts, emphasizing that raw frequency comparisons alone can be misleading without attention to dispersion, function, and context. Castello [14] extends the study of stance to adverbial markers in L1 and L2 spoken elicited conversations, showing that L2 speakers tend to employ a narrower repertoire of stance resources than L1 speakers.

The present study builds on and complements these contributions in four specific ways. First, whereas Fortanet-Gómez [48] analyses L1 lectures, we extend that line to an L1 vs. L2 contrast specifically in faculty-level research discourse. Second, whereas Gablasova et al. [49] provide a methodological framework, we apply their cautions concretely by triangulating frequency with phraseology and discourse function. Third, whereas Castello [14] analyses adverbial stance in elicited undergraduate interaction, we analyse lexical verb hedges in unelicited faculty-level research presentations. Fourth, and most importantly, we move beyond description to design: we deliver a full “diagnosis-to-treatment” pipeline, including a replicable DDL module and sample worksheets, so that corpus evidence can be directly actionable in the EAP classroom. Taken together, these four features define the specific contribution of the present study. To the best of our knowledge, no previously published corpus study documents the unelicited spoken academic English of faculty-level Chinese scholars, examines lexical verb hedges at the phraseological level in such data, or derives classroom-ready teaching materials directly from the resulting diagnosis. It is within this specific empirical and applied space — rather than in the broader, well-cultivated field of stance studies as a whole — that the present study claims its original contribution.

## 3. Research design and methodology

Ethics statement. This study is based entirely on the analysis of publicly available secondary data. The Chinese Scholars’ Academic Spoken English Corpus (CASEC) was compiled from open-access online resources (public academic lecture recordings, open-access international conference presentations, and publicly defended PhD dissertations available through institutional repositories and public video platforms), and the Michigan Corpus of Academic Spoken English (MICASE) is a publicly accessible, published corpus. The study did not recruit human participants, did not involve direct interaction or intervention, and did not handle any personal identifying information beyond speakers’ public professional identity in their public academic role. The research protocol was reviewed by the Ethics Review Committee of the School of Foreign Languages, Dalian Maritime University, which confirmed in writing that the study qualifies as secondary analysis of publicly available data and accordingly granted a formal waiver of ethical approval; for the same reason, the requirement for informed consent (written or verbal) was also formally waived.

### 3.1 Corpus compilation and data sources

This study employs a corpus-based Contrastive Interlanguage Analysis (CIA) approach [50], comparing an L2 scholar corpus with a documented reference corpus representing the Anglo-American academic spoken norm.

#### 3.1.1 The L2 scholar corpus: CASEC.

The Chinese Scholars’ Academic Spoken English Corpus (CASEC) is a specialized L2 expert corpus compiled specifically for this research project. Throughout this paper, we use the term “L2 scholar corpus” (rather than “learner corpus”) to acknowledge that the speakers represented in CASEC are not learners in the usual sense: they are active faculty members, postdoctoral researchers, or advanced doctoral candidates who already use English for professional research purposes, and who are pragmatically expanding — rather than initially acquiring — their academic English repertoire.

The compilation process spanned seven years, from 2018 to 2025, ensuring the data reflects current usage trends. The corpus totals 696,009 words and comprises 279 audio-recorded events. The data sources include academic lectures, international conference presentations (including Q&A sessions), and PhD dissertation defenses.

Strict selection criteria were applied:

(1) Speaker Profile. All speakers are native Chinese speakers (L1 Chinese) who are active scholars (faculty members, postdoctoral researchers, or advanced doctoral candidates) in mainland China universities.
(2) Language. The medium of instruction or presentation was exclusively English.
(3) Discipline. The corpus covers a balanced range of disciplines, broadly categorized into STEM (Science, Technology, Engineering, Mathematics) and HSS (Humanities and Social Sciences), to minimize disciplinary bias.
(4) Source licensing. Only open-access recordings whose licensing permits non-commercial research use were included.

The disciplinary and genre-level composition of CASEC is summarized in Table 1.

**Table 1:** Disciplinary and genre-level composition of CASEC.

Genre	STEM Events	HSS Events	Total Events	Tokens	Duration (h)
Academic Lectures	62	58	120	321,451	~52
Conference Presentations (incl. Q&A)	47	41	88	207,283	~31
PhD Dissertation Defenses	36	35	71	167,275	~25
**Total**	**145**	**134**	**279**	**696,009**	**~108**

Note. STEM = Science, Technology, Engineering, Mathematics; HSS = Humanities and Social Sciences. Token counts are based on the post-cleaning, English-only transcript. Duration is approximate.

#### 3.1.2 The reference corpus: MICASE.

The Michigan Corpus of Academic Spoken English (MICASE) serves as a documented reference corpus representing the Anglo-American academic spoken norm. MICASE contains 1,848,364 words across 152 events recorded at the University of Michigan [51]. We selected MICASE — rather than, for example, the English as a Lingua Franca in Academic Settings (ELFA) corpus [52] — for three principled reasons that align with our specific pedagogical aim.

First, the goal of this study is not to measure “ELF success” in Chinese scholars’ output, but to help Chinese scholars expand their rhetorical repertoire to include the specific stance resources that still dominate the Anglo-American gatekeeping channels (SSCI/SCI journals, high-impact international conferences, and research-grant-committee review) through which many of them must pass for career progression under the “Double First-Class” initiative. MICASE documents authentic spoken academic English at a top U.S. research university and is therefore a more appropriate reference for this specific aim. Second, MICASE has been extensively used and scrutinized in more than two decades of published research [9,18,48,53], giving us strong comparability with prior studies. Third, MICASE shares genre characteristics with CASEC (lectures, defenses, seminars), allowing for broadly like-for-like comparison.

We explicitly acknowledge — and have integrated this acknowledgement into our discussion and limitations sections — that the use of MICASE as a reference point does not imply a normative claim that L1 Anglo-American usage is inherently “correct” or that Chinese scholars should aspire to converge on it. Rather, we view MICASE as one among several relevant reference points; an ELFA-based comparison would address a different and equally important question (“how do Chinese ELF speakers differ from other ELF speakers?”) which we identify as a priority for future work (see Section 6).

The design profile of ELFA itself clarifies why the two corpora answer different questions. ELFA, whose compilation was completed in 2008, comprises approximately one million words of academic speech recorded at Finnish universities, produced by some 650 speakers from 51 first-language backgrounds [52]. It is an authoritative record of English as an academic contact language in Northern-European university settings. Precisely for this reason, however, benchmarking CASEC against ELFA would address how Chinese scholars pattern relative to a heterogeneous, largely Europe-based ELF population — a valuable question, but a different one from that motivating this study — and would implicitly install that population’s usage as the pedagogical reference point, a normative move no less contestable than the one it would replace. Nor would the substitution improve recency, since the ELFA recordings predate CASEC (2018–2025) by a margin at least as large as that of MICASE.

To make the inferential scope of the study fully explicit: the quantitative contrasts reported in Section 4 license conclusions only about differences between CASEC and this documented Anglo-American reference, and the pedagogical implications developed in Section 5 are confined to contexts in which Anglo-American conventions continue to operate as gatekeeping norms. The study advances no claims about the communicative success or failure of Chinese scholars in ELF interaction; evaluating such claims would require an ELF reference corpus such as ELFA, and we return to this point in Section 6.1.

The temporal gap between the two corpora is worth noting; however, the core genre conventions of academic presentations are relatively stable, providing a valid baseline for this exploratory contrastive analysis [53]. Both corpora were transcribed and contain metadata on the speaker (e.g., academic status) and event (e.g., discipline, genre), allowing for robust comparative analysis. The basic quantitative parameters of the two corpora used in this study are summarized in Table 2.

**Table 2:** Basic information of the two corpora.

Corpus	Events	Tokens	Duration (h)	Reference
**CASEC**	279	696,009	~108	This study
**MICASE**	152	1,848,364	~190	Simpson et al. [51]

Table 3 summarizes the MICASE genre categories and their mapping to the three CASEC sub-genres. Three CASEC sub-genres map cleanly to MICASE categories; some MICASE categories (e.g., Advising Sessions) have no CASEC counterpart and are noted as such so the reader can assess the comparability of any cross-corpus claim.

**Table 3:** Mapping of MICASE genre categories to CASEC sub-genres.

MICASE genre category	Corresponding CASEC sub-genre	MICASE tokens (approx.)
Large/ Small Lectures; Colloquia	Academic Lectures	~1,020,000
Student Presentations	Conference Presentations	~310,000
Dissertation Defenses	PhD Dissertation Defenses	~165,000
Advising Sessions; Service Encounters; Tours	No direct counterpart in CASEC	~353,000

Note. Token counts for MICASE are approximated from the official MICASE metadata documentation (Simpson et al. [51]). Rows without CASEC counterparts were excluded from sub-genre-level comparisons; they are retained in whole-corpus normalization as standard in prior MICASE-based studies.

#### 3.1.3 Transcription procedure and conventions.

To ensure methodological transparency, we describe the full transcription pipeline for CASEC here and provide the complete convention sheet in the S1 File. The pipeline consists of five sequential stages, summarized in Table 4.

**Table 4:** The five-stage CASEC transcription pipeline.

Stage	Procedure	Tool/ Software	Personnel
1	Automatic transcription	OpenAI Whisper large-v3	Automated
2	Manual correction against audio	ELAN v6.7	Two graduate RAs
3	Independent verification	ELAN v6.7	Senior researcher
4	Application of transcription conventions	MICASE conventions (adapted)	All three transcribers
5	POS tagging; concordance preparation	CLAWS7 via CQPweb; AntConc v4.2	Lead author

(i) Automatic transcription. Each audio recording was first passed through the OpenAI Whisper large-v3 automatic speech recognition model (English-language setting), producing an initial time-aligned draft transcript.
(ii) Manual correction. The draft transcript was manually corrected against the original audio in ELAN (version 6.7) by two trained research assistants who are advanced graduate students in applied linguistics. Corrections covered lexical accuracy, speaker attribution, and the insertion of paralinguistic features.
(iii) Verification. A third researcher independently verified 100% of the corrected transcripts against the audio, focusing on potentially ambiguous stretches (overlapping speech, accented realizations of target lexical items).
(iv) Conventions. Transcription conventions broadly followed the MICASE conventions (e.g., (.) for short pauses, < laugh> for laughter, [word] for uncertain stretches, – for truncated words), with minor local adaptations documented in the S1 File.
(v) Reliability. Inter-transcriber agreement was assessed on a 10% random sub-sample using a word-level edit-distance metric, yielding a mean agreement rate of 98.4%. Residual discrepancies were resolved by discussion among the three researchers. Final transcripts were part-of-speech tagged with CLAWS7 via the CQPweb interface prior to AntConc-based concordance analysis.

### 3.2 Data analysis procedures

The analysis followed a rigorous, multi-step process designed to move from quantitative frequency to qualitative interpretation.

#### Step 1: Selection of target verbs.

Based on Vass’s [19] comprehensive study on epistemic verbs in discourse, a candidate list of 10 high-frequency lexical verbs with strong potential for hedging functions was compiled. This list includes verbs of perception, speculation, and evaluation (e.g., see, think, assume, consider, evaluate, indicate, look, feel, observe), shown in Table 5. These verbs were chosen because they represent the most common lexical means of expressing stance in spoken English.

**Table 5:** Candidate list of lexical verb hedges (adapted from Vass [19]).

Category	Lexical Verb Hedges
**Perceptual**	see, feel, look
**Speculative**	think (speculative), assume, consider
**Quotative**	think (quotative), indicate (quotative)
**Evaluative**	evaluate, observe

#### Step 2: Data retrieval and coding.

Using the concordancing software AntConc (Version 4.2), all inflected forms of the 10 target verbs were retrieved from both CASEC and MICASE, generating a total dataset of 9,567 concordance lines. Manual disambiguation was critical, as the hedging function is highly context-dependent. For instance, the verb think can function as a cognitive process (“I think about the problem”) or as a hedge (“I think this is valid”) [54]. Two researchers manually coded all instances. An instance was coded as a “hedge” only if it was used to “modulate the speaker’s commitment to the truth of a proposition” or to soften an assertion. Inter-coder reliability was calculated on a 20% random sample, resulting in a Cohen’s Kappa score of 0.85, indicating high agreement. Discrepancies were resolved through discussion with a third senior researcher.

#### Step 3: Frequency and significance testing.

All raw frequencies were normalized to a common basis (per million words, pmw) to account for the different sizes of the two corpora (see Table 2). The Log-likelihood (LL) statistic was calculated to determine the statistical significance of any frequency differences, as it is more robust than Chi-square for corpora of different sizes. A p-value of < 0.05 was set as the threshold for significance [55].


$$\begin{array}{c} LL=\text{2}\times \Sigma O_{i}ln(O_{i}/E_{i}) \end{array}$$

where Oᵢ represents the observed frequency and Eᵢ represents the expected frequency.

#### Step 4: Phrasal pattern analysis.

To move beyond single words and capture the “units of meaning” [56], the analysis focused on recurrent lexical sequences or “phrasal patterns” anchored by the 10 target verbs. Following Hunston and Francis [54], we define a phrasal pattern as a recurrent lexico-grammatical frame consisting of a pivot verb and the lexico-grammatical slots that immediately surround it (typically its grammatical subject and its complement). We operationalized this definition in three steps:

(a) For each of the 10 target verbs, we extracted the full concordance (± 8 words from the node) from both corpora.
(b) Every instance previously identified as a hedge (Step 2) was re-coded along two phraseological dimensions: (i) grammatical subject type (first-person singular I, first-person plural we, anticipatory it, inanimate noun-phrase, or other); and (ii) complement type (finite that-clause, zero complement, non-finite complement, or other).
(c) Cross-tabulating these two dimensions yielded the candidate phrasal patterns. Patterns were retained for further analysis if they were attested by at least 50 raw tokens in at least one of the two corpora. Four patterns met this criterion: I V (that), We V (that), It V (that), and N-inanimate V (that).

Table 6 summarizes the four major phrasal patterns, their operational definitions, and illustrative examples from the two corpora.

**Table 6:** Operationalisation of the four phrasal patterns, with illustrative examples.

Pattern	Subject type	Complement type	CASEC example	MICASE example
**I V (that)**	1st p. singular	that/ zero	*“I think that is one of the limitations here.”*	*“I think you’ll enjoy that, it is a very interesting perspective.”*
**We V (that)**	1st p. plural	that/ zero	*“From this chart, we can see EU is an important source.”*	*“We find that the effect is robust across conditions.”*
**It V (that)**	Anticipatory it	that/ to-infinitive	*“It seems that this problem has been addressed.”*	*“It looks like we’re running out of time.”*
**N-inanimate V (that)**	Inanimate NP	that/ zero	*“The data indicate that this approach is effective.”*	*“The results suggest a clear pattern.”*

These four patterns then served as the primary units for the qualitative discourse analysis reported in Section 4.2 [57].

**Fig 1:**
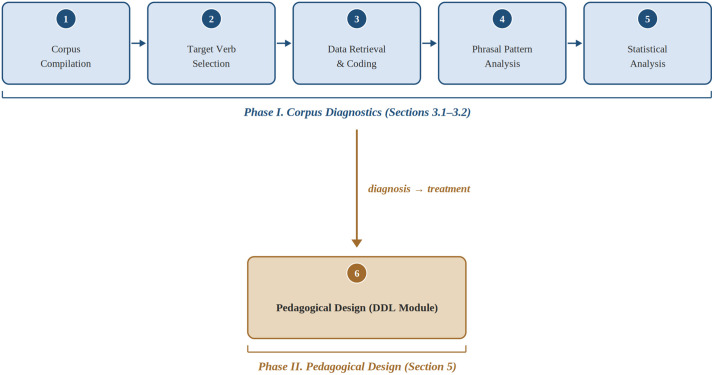
The six-step analytical pipeline. Steps 1–5 (Phase I) cover the corpus-diagnostic procedures detailed in Sections 3.1–3.2 — Corpus Compilation → Target Verb Selection → Data Retrieval & Coding → Phrasal Pattern Analysis → Statistical Analysis. Step 6 (Phase II) corresponds to the pedagogical module presented in Section 5 (Pedagogical Design/ DDL Module).

Fig 1 provides a visual recap of the full six-step analytical pipeline. Steps 1–5 correspond to the corpus-diagnostic procedures detailed in the previous two sub-sections; Step 6 corresponds to the pedagogical module presented in Section 5.

## 4. Results and discussion: Diagnosing pedagogical targets

### 4.1 Overall distribution of lexical verb hedges

As shown in Table 7, a significant difference emerged in the overall frequency of lexical verb hedges. Chinese scholars (CASEC) used these devices at a normalized frequency of 3,592 pmw, which was significantly lower than the 3,823 pmw observed in the reference corpus (MICASE) (LL = –7.27, p < 0.01). This macro-level finding suggests a general underuse of this specific stance-marking resource by the Chinese scholars.

**Table 7:** Overall frequency of lexical verb hedges.

Corpus	Raw Freq.	Norm. Freq. (pmw)	Log-likelihood	p-value
**CASEC**	2,500	3,592	–7.27	0.007
**MICASE**	7,067	3,823	—	—

However, this aggregate figure masks a much more complex picture, revealed in the breakdown by individual verbs (Table 8). The MICASE speakers showed a profound reliance on think (totaling 2,803 pmw) as their primary hedging resource. In contrast, Chinese scholars significantly underused think (1,247 pmw). Strikingly, CASEC speakers compensated for this underuse by significantly overusing a range of other verbs. The most dramatic overuse is with see, which is roughly three times more frequent in CASEC (1,027 pmw) than in MICASE (310 pmw). This is followed by significant overuse of assume, consider, evaluate, and indicate.

**Table 8:** Frequencies of specific lexical verb hedges (pmw).

Lexical Verb Hedge	CASEC (pmw)	MICASE (pmw)	Log-likelihood	p-value
*see*	1,027	310	+635.91	<0.001
*think (speculative)*	1,122	2,359	–431.23	<0.001
*assume*	283	116	+77.79	<0.001
*consider*	253	93	+83.84	<0.001
*evaluate*	148	12	+164.78	<0.001
*feel*	145	209	–11.36	<0.001
*think (quotative)*	125	444	–176.40	<0.001
*indicate (quotative)*	121	23	+84.22	<0.001
*observe*	95	10	+95.32	<0.001
*look*	93	248	–68.99	<0.001

This divergence suggests a potential difference in the underlying cognitive metaphor for knowledge construction [38]. The MICASE preference for think aligns with a “mentalistic” model (“Knowledge is a product of thought”), where knowledge is framed as a product of individual cognition and assessment. The CASEC preference for see may reflect a “visualized” or “embodied” cognitive model (“Knowledge is seeing”) [26]. By framing an argument as something that can be “seen” (e.g., “We can see the result”), the speaker presents the claim as an objective, shared observation rather than a subjective opinion. While potentially intended as a strategy of modesty (avoiding personal opinion), it can paradoxically be perceived as more assertive by effacing the individual author’s role in interpretation and presenting the finding as a self-evident fact [58].

### 4.2 Phrasal pattern analysis: The ‘I’ vs. ‘We’ distinction

The analysis of phrasal patterns (Table 9) reveals the most critical finding of the study. The MICASE speakers used the first-person singular pattern I V (that) (e.g., I think, I feel, I assume) at an overwhelmingly high frequency (2,126 pmw). This pattern, which explicitly marks an individual authorial stance, was the default strategy in the reference corpus. In stark contrast, this pattern was exceptionally rare in the CASEC data (76 pmw). Conversely, Chinese scholars showed a massive preference for the first-person plural pattern We V (that) (e.g., We see, We assume), using it at 889 pmw, compared to only 87 pmw in MICASE. This stark I vs. We dichotomy is the central diagnostic finding of this study and warrants detailed qualitative examination.

**Table 9:** Frequencies of the four major phrasal patterns (pmw).

Phrasal Pattern	CASEC (pmw)	MICASE (pmw)	Log-likelihood	p-value
***I V (that)***	76	2,126	–2,086.17	<0.001
***We V (that)***	889	87	+915.93	<0.001
*It V (that)*	46	29	+4.24	0.039
*N-inanimate V (that)*	217	61	+103.29	<0.001

#### 4.2.1 The I V (that) pattern: Individual stance and negative politeness.

In Anglo-American academic discourse, the I V (that) pattern is a primary strategy for constructing an individualized authorial identity [1,59]. It is the tool by which a scholar explicitly takes responsibility for their claims. As demonstrated in the corpus examples below, it functions as a crucial negative politeness strategy.

Consider example (1), drawn from MICASE:

(1) *I think* you’ll enjoy that, it is a very interesting perspective. **(MICASE, COL485JU098)**

Here, the hedge I think softens the directive force of the recommendation, respecting the audience’s autonomy to disagree. Similarly, in critical contexts, speakers often use I don’t think… to effectively mitigate the Face-Threatening Act (FTA) of challenging a colleague’s argument [29], as illustrated in example (2):

(2) *I don’t think* that’s quite the right way to look at the problem, actually. **(MICASE, DIS405SG069)**

By attributing the judgment to the speaker’s subjective assessment rather than presenting it as an objective fact, the speaker leaves room for debate. This explicit marking of subjectivity and individual responsibility is a cornerstone of Anglo-American academic persuasion [10]. The significant underuse of this pattern by Chinese scholars suggests a critical gap in their rhetorical repertoire, potentially making their arguments sound less nuanced or overly detached.

#### 4.2.2 The we V (that) pattern: Collective stance and positive politeness.

In contrast, the CASEC speakers’ heavy reliance on We V (that) reflects a different rhetorical orientation. This pattern, particularly the high-frequency We see, aligns with a positive politeness strategy [48], as illustrated in examples (3) and (4):

(3) From this chart, *we can see* EU is an important source for our exports. **(CASEC, CONF_042)**
(4) *We assume* here that the two variables are independent. **(CASEC, DEF_017)**

In example (3), the use of we is “inclusive,” inviting the audience to join the speaker in a shared observation. This strategy builds common ground and establishes a sense of community, aligning with Chinese cultural scripts valuing collective harmony [25,60]. In example (4), we assume diffuses the responsibility for a methodological choice, presenting it as a shared premise of the research community rather than an individual’s decision. While this strategy is effective in creating solidarity, its overuse can obscure the author’s unique contribution and voice [61].

### 4.3 Discussion: Epistemological stance and pragmatic mismatch

This systematic I vs. We distinction represents more than just a stylistic preference; it reveals a fundamental pragmatic mismatch rooted in differing epistemological stances. The We-strategy, combined with the “visual” metaphor of see and the overuse of depersonalized It/ N-inanimate subjects [62], reveals a coherent rhetorical style: one that prioritizes a collective, objective, and depersonalized presentation of knowledge. This reflects an epistemology where “truth” is external and observable, rather than internal and constructed.

While this style is effective in many contexts, its overuse by Chinese scholars is problematic in Anglo-American academic venues for several reasons. First, the overuse of we may function as a strategy to diffuse responsibility, masking a lack of individual authorial confidence [59]. Many Chinese scholars equate academic objectivity with personal detachment, mistakenly believing that avoiding I makes their research appear more scientific. Second, an over-reliance on inclusive we can be misinterpreted as presumptuous (assuming the audience’s agreement) or overly didactic. Third, and most importantly, the absence of the I-pattern means the speaker lacks a primary L2 tool for tactfully advancing a novel or critical argument. In Anglo-American academia, the I is often the symbol of critical thought and intellectual ownership [63]. A speaker who avoids I may be perceived as lacking a critical stance, which can negatively impact their reception in international conferences or collaborative projects.

Furthermore, this finding has implications for the ongoing debate between English as a Foreign Language (EFL) and English as a Lingua Franca (ELF). While ELF researchers [27] argue for the acceptance of local varieties, in high-stakes gatekeeping contexts (such as publication and grant review), access to the conventions still dominant in Anglo-American gatekeeping venues is often required [64]. We wish to be explicit that our position is not one of native-speakerism. The pedagogical goal of the intervention we propose is not to eradicate the We-pattern or to enforce L1 usage; it is to expand Chinese scholars’ rhetorical repertoire so that they can strategically deploy both I-based and We-based resources according to the specific communicative goal of a given context. If this “pragmatic ossification” is not addressed, Chinese scholars risk remaining on the periphery of Anglo-American-dominated academic dialogue, unable to fully engage in the crucial interpersonal negotiations that define successful scholarship in those venues.

## 5. Pedagogical application: A proposed DDL module for pragmatic intervention

The corpus diagnostics reveal a clear pedagogical gap: Chinese scholars tend to over-rely on collective stances while underutilizing individualized hedges. To address this, we propose a “diagnosis-to-treatment” approach, translating the quantitative findings into a concrete Data-Driven Learning (DDL) module.

### 5.1 Rationale and learning objectives

This module moves beyond traditional, prescriptive EAP instruction. Instead, it aligns with a DDL approach by empowering participants to discover pragmatic functions themselves through exposure to authentic corpus data [40]. We carefully distinguish between the L2 scholars whose discourse is represented in CASEC (the diagnostic population) and the participants in the DDL module (the pedagogical audience). The learning objectives for the latter are:

- Awareness (Noticing). Participants will be able to identify I V (that) and We V (that) as distinct phrasal patterns for academic stance-marking.
- Analysis (Hypothesizing). Participants will articulate the different pragmatic functions of each pattern (e.g., I think for mitigating personal/critical claims; We see for building consensus).
- Application (Practice). Participants will strategically select and use the appropriate pattern in simulated academic speaking tasks based on their specific rhetorical goal [44].

### 5.2 Sample DDL module: “Authorial voice: ‘I’ vs. ‘We’ in academic presentations”

This module is designed as a 90-minute workshop, structured in four phases. Complete, classroom-ready versions of all materials — Worksheet A (Individual Stance), Worksheet B (Collective Stance), the Instructor’s Guiding-Question Sheet, and the Role-Play Scenario Cards — are provided as a separate supplementary file (S2 File).

#### Phase 1: Observation (Worksheets).

Participants are divided into groups and given two worksheets containing authentic concordance lines, selected to maximize contrast.

- Worksheet A (Individual Stance — from MICASE) presents 20 concordance lines featuring I think…, I feel…, I assume… (see S2 File for the full sheet).
- Worksheet B (Collective Stance — from CASEC and MICASE) presents 20 concordance lines featuring We see…, We find…, We assume… (see S2 File).

The instructor prompts participants with the guiding questions in S2 File, for example:

(1) In Worksheet A, does the speaker sound 100% certain, or are they leaving room for doubt?
(2) Why does the expert speaker say I think this is wrong instead of just This is wrong? How does this affect the listener’s feeling?
(3) In Worksheet B, who exactly does we refer to? Is it the research team, or the speaker plus the audience?

#### Phase 2: Hypothesis and generalisation.

The instructor leads a plenary discussion to map forms to functions. The class is guided to conclude that I V (that) is often used for “Expressing personal opinion” and “Softening criticism” (Negative Politeness), while We V (that) is used for “Inviting agreement” and “Presenting shared data” (Positive Politeness). The instructor explicitly introduces the concept of pragmatic mismatch, discussing how avoiding I might be perceived in international contexts (e.g., sounding evasive or lacking original contribution).

#### Phase 3: Practice and application.

Participants engage in role-play scenarios (S2 File) to practise context-sensitive choices.

- Scenario 1 (The Polite Critic). Participants must criticize a well-known theory during a Q&A session. They are instructed to avoid direct confrontation. Target usage: I feel that…, I think….
- Scenario 2 (The Data Presenter). Participants must present non-controversial results from a chart to a general audience. Target usage: We can see…, The data shows….

Groups perform short presentations, receiving peer feedback on whether their stance markers were appropriate for the communicative goal.

#### Phase 4: Reflection.

To consolidate learning, participants write a short reflection log (200 words) on how this awareness of I vs. We patterns will alter their future presentation strategies, specifically addressing their own previous habits [65].

## 6. Conclusion

This study executed an integrated research programme, moving from a corpus-based diagnosis of a specific pragmatic feature to the design of a targeted pedagogical intervention. The contrastive analysis identified a systematic divergence: Chinese scholars’ spoken academic English was characterized by a significant underuse of I-based individual stance patterns and a heavy overuse of We-based collective patterns. Theoretically, this study interprets this distinction not as a simple error, but as a systematic difference in rhetorical strategy, likely rooted in the pragmatic transfer of L1 cultural scripts related to politeness and modesty.

While the We-pattern serves a valid positive politeness function, its overuse represents a significant gap in the pragmatic repertoire of Chinese scholars relative to the Anglo-American venues in which many of them must operate. This mismatch can hinder their rhetorical effectiveness in Anglo-American academic settings, where the explicit marking of an individual, critical stance is highly valued. The primary contribution of this research is the demonstration of a “diagnosis-to-treatment” pipeline. By identifying a precise pedagogical need, we designed a specific DDL module — including full classroom-ready worksheets — that leverages guided induction to help scholars expand their strategic options. This work answers the call for a more symbiotic relationship between corpus-based L2 research and EAP pedagogy.

### 6.1 Limitations and future research

Several limitations of this study point to productive directions for future work. First, our use of MICASE as the sole reference corpus, though principled in relation to our aim (equipping Chinese scholars for Anglo-American gatekeeping venues), inevitably foregrounds a particular subset of global academic norms. A complementary study comparing CASEC against the ELFA corpus [52] would allow us to ask the distinct and equally important question of how Chinese scholars’ spoken output patterns against other ELF speakers, and we have identified this CASEC–ELFA triangulation as the immediate next phase of this research programme, to be reported as a stand-alone study. Second, there is a temporal gap between the MICASE recordings and CASEC; although the core genre conventions of academic presentations are relatively stable [53], a more recent L1 reference dataset would further strengthen confidence in the cross-corpus contrast. Third, the DDL module proposed here has been designed but not yet empirically tested; a classroom-based intervention study, fully closing the “diagnosis-to-treatment” loop, is the natural next step. Fourth, we have focused on ten high-leverage lexical verb hedges; extending the analysis to modal verbs, adverbial stance markers, and booster-hedge interaction would yield a more complete picture of the pragmatic repertoire.

## Supporting information

S1 File Corpus data and concordances (English-language version). A compressed file containing the complete dataset and analysis details underlying the study, organized into two folders: (i) CASEC — all lists and concordances of hedges in CASEC; (ii) MICASE — all lists and concordances of hedges in MICASE. All file names, folder names, and metadata have been prepared in English in accordance with PLOS ONE policy.
(ZIP)

S2 File Sample DDL Worksheets. Worksheet A, Worksheet B, Instructor’s Guiding-Question Sheet, and Role-Play Scenario Cards for classroom use.
(DOCX)
