# Supplementary material for: Bridging corpus diagnostics and EAP pedagogy: A corpus-driven study on lexical hedges in Chinese scholars’ spoken academic English
Source: PLoS One. 2026 Jul 6;21(7):e0353106. doi: 10.1371/journal.pone.0353106 (PMC13336206; doi:10.1371/journal.pone.0353106)
Supplement: S2 File — Worksheet A, Worksheet B, Instructor’s Guiding-Question Sheet, and Role-Play Scenario Cards for classroom use. (DOCX) [file pone.0353106.s002.docx]

S2 File. Sample DDL Worksheets

Standalone classroom-ready materials corresponding to Appendix A of the manuscript.

# Appendix A. Sample DDL Worksheets

This appendix presents the full, classroom-ready materials referenced in Section 5.2. An editable standalone version is also available as S2 File.

## A.1 Worksheet A — Individual Stance (MICASE Concordance Lines)

Instructions for participants. Read the concordance lines below carefully. For each line, identify (a) what the speaker is doing (informing, criticising, recommending, softening a disagreement, etc.) and (b) how the italicised hedging expression affects the force of the utterance. Be prepared to share your answers in the plenary.

(1) …well, I think you'll enjoy that, it is a very interesting perspective on the issue…

(2) …I don't think that's quite the right way to look at the problem, actually…

(3) …and I feel like the data here could be read in more than one way…

(4) …I assume most of us would agree that the baseline needs to be reconsidered…

(5) …um, I think what we really want to ask is whether this generalises at all…

(6) …I feel it's important to acknowledge that we're making several simplifying assumptions…

(7) …I think the author is probably right, but maybe for different reasons than she gives…

(8) …I assume you've already controlled for that, but just to be safe…

(9) …I don't feel comfortable calling this a causal effect, at least not yet…

(10) …I think, actually, the more interesting question is what happens at the tail…

(11) …I feel we need to be a bit more cautious about extrapolating these results…

(12) …I assume the audience is familiar with the basic framework so I'll go quickly…

(13) …I think, on balance, the evidence is in favour of the first interpretation…

(14) …I don't think we can really claim that, at least not without more data…

(15) …I feel that the authors have somewhat overstated their conclusions here…

(16) …I think it's fair to say that the literature is genuinely split on this…

(17) …I assume that, if we had more time, we'd want to examine the subgroups…

(18) …I think, you know, the picture is more complicated than the abstract suggested…

(19) …I feel a little uneasy about that move, to be honest…

(20) …I think the key point, and I might be wrong, is the interaction term…

## A.2 Worksheet B — Collective Stance (CASEC and MICASE Concordance Lines)

Instructions for participants. The concordance lines below all feature a first-person-plural subject. For each line, identify (a) who exactly "we" refers to (e.g., the research team, the speaker plus audience, the research community, humanity in general) and (b) whether a first-person-singular alternative (I see / I find / I assume) would have been more or less effective, and why.

(1) …so, from this chart, we can see that EU is an important source for our exports…

(2) …we find that the effect is robust across conditions…

(3) …we assume here that the two variables are independent…

(4) …and, as we can see, the curve peaks at around the three-month mark…

(5) …we observe a clear downward trend in the second half of the period…

(6) …if we consider the left panel, we can see that…

(7) …as we know from previous studies, the baseline rate is around 15 per cent…

(8) …we assume, for the sake of this analysis, that the sample is representative…

(9) …we can clearly see that the intervention group outperforms the control…

(10) …we believe this approach opens up several new research questions…

(11) …we may conclude that the hypothesis is supported by the data…

(12) …here we see the same pattern repeating itself across disciplines…

(13) …we find, somewhat surprisingly, that the reverse effect also holds…

(14) …we consider this to be a reasonable approximation, given the constraints…

(15) …we evaluate the model using three standard benchmarks…

(16) …we think this result has important implications for policy…

(17) …let us see what happens if we relax this assumption…

(18) …we look at the distribution of responses across the three groups…

(19) …we assume the reader is familiar with the basic structure…

(20) …we can see, therefore, that the two explanations are not mutually exclusive…

## A.3 Instructor's Guiding-Question Sheet

Below are suggested questions to structure the plenary discussion at the end of Phase 1 and during Phase 2. Instructors are encouraged to adapt them to their specific audience.

Q1. On Worksheet A, look at lines (1), (2), and (14). Does the speaker sound completely certain, or are they leaving room for doubt? Who carries the responsibility for the claim — the speaker alone, or "everyone"?

Q2. On Worksheet A, compare lines (2) and (9). Both are challenging someone else's view. How does the hedge change the social effect of the challenge?

Q3. On Worksheet B, line (1) says we can see, but only one person (the speaker) is actually looking at the chart. Who, then, does we refer to? Why is it useful to include the audience in the "seeing"?

Q4. On Worksheet B, line (3) says we assume. If the speaker were the only person who made this assumption, would I assume sound different? Better? Worse?

Q5. Imagine you have to disagree with a senior colleague during a Q&A. Which pattern would you pick — I think or We think — and why?

Q6. Imagine you are presenting an uncontroversial result from your joint paper. Which pattern would you pick and why?

Q7. What risks might come from always using we and never using I? What risks from always using I and never using we?

Q8. Can you think of a situation in your own field where using we would be seen as presumptuous? A situation where using I would be seen as self-centred? How do you decide?

## A.4 Role-Play Scenario Cards (Phase 3)

Each participant (or pair) is given one of the four cards below, prepares for 10 minutes, and then delivers a 2–3 minute spoken mini-presentation or Q&A turn. Observers note which stance patterns were used and whether they matched the communicative goal.

Card 1 — The Polite Critic.

You are attending an international conference in your discipline. The keynote speaker has just argued that a widely-accepted theory in your field is fundamentally flawed. During the Q&A, you strongly disagree but you do not want to come across as aggressive. You also want to preserve the possibility of future collaboration with the speaker. Prepare a 90-second spoken response. Target stance pattern: I V (that) (e.g., I think, I feel, I'm not entirely sure…).

Card 2 — The Data Presenter.

You are presenting joint results from your research team's latest experiment at a large international conference. The findings are uncontroversial and the audience broadly includes researchers from adjacent fields. You have 2 minutes and a single chart. Prepare a spoken walk-through of the chart. Target stance pattern: We V (that) (e.g., we can see, we find, we observe…).

Card 3 — The Doctoral Candidate Under Questioning.

You are defending your PhD. A committee member challenges the methodological decision at the heart of your dissertation, implying that another choice would have been superior. You must (a) acknowledge the validity of the challenge and (b) defend your own decision without alienating the committee. Prepare a 90-second response. Target stance patterns: a strategic mix of I V (that) and We V (that).

Card 4 — The Grant Interview.

You are being interviewed by an international funding panel. A panellist asks what is genuinely novel about your proposal. You need to claim intellectual ownership of the novelty without sounding boastful, and without hiding behind the collective. Prepare a 90-second spoken pitch. Target stance patterns: predominantly I V (that) for the novelty claim; We V (that) where describing your team's prior work.
